# Disaster medicine in Swedish undergraduate medical education: analysing current programs and future integration in the six-year curriculum

**DOI:** 10.1186/s12909-025-07324-2

**Published:** 2025-05-20

**Authors:** Sofia Olsson, Lisa Kurland, Fabian Taube, Joakim Björås, Yohan Robinson

**Affiliations:** 1 Centre for Disaster Medicine, University of Gothenburg, Gothenburg, Sweden; 2 School of Medical Sciences, Örebro University, Örebro, Sweden

**Keywords:** Medical programs, Disaster medicine, Disaster preparedness, Curriculum development, Swedish medical education

## Abstract

**Background:**

Disaster medicine involves managing situations where medical needs exceed available resources. In Sweden, disaster medicine is not yet a mandatory component of the medical education. Since the introduction of a revised six-year medical curriculum in 2021, it is unclear how disaster medicine will be integrated into the new program. This study aimed to evaluate the status of undergraduate disaster medicine education in Swedish medical schools, the teaching methodologies employed, plans for future curriculum integration, and the extent of variation across universities.

**Methods:**

We conducted a comprehensive review of syllabi from all Swedish medical programs to identify the inclusion of disaster medicine. Additionally, semi-structured interviews were conducted with 13 representatives from all seven Swedish medical schools, including those responsible for disaster medicine education or members of the education boards. The interviews explored teaching methods, curriculum content, and plans for the new six-year program. Data were analysed using qualitative content analysis.

**Results:**

Disaster medicine is included in the curriculum of all Swedish medical programs; however, its content, extent, and teaching approaches vary. Lectures are the most common teaching method, with some schools incorporating case discussions, tabletop exercises, and disaster simulations. Most medical faculties plan to maintain or expand their disaster medicine curriculum. However, there is no formal collaboration between universities in developing or standardizing disaster medicine education for the new curriculum.

**Conclusion:**

The current level of disaster medicine education in Swedish medical schools requires enhancement in both quality and scope. Variations between universities would need to be minimized to ensure a more consistent approach. Preliminary plans for the new six-year medical program suggest that disparities between universities may persist, underscoring the need for a coordinated effort in standardizing disaster medicine education at the undergraduate level.

**Supplementary Information:**

The online version contains supplementary material available at 10.1186/s12909-025-07324-2.

## Introduction

Disaster medicine is increasingly recognized as a distinct academic discipline that encompasses the medical, organizational, and ethical aspects of providing care in situations where needs exceed available resources [1, 2]. It is grounded in principles such as triage, surge capacity, coordination, and crisis leadership, and intersects with public health, emergency medicine, and humanitarian response [3].

Disasters can be natural, such as the 2023 earthquakes in Syria and Turkey or man-made, such as the ongoing humanitarian crisis in Gaza. Sweden has also faced disasters over the years, such as the 1994 maritime disaster involving the passenger ferry Estonia, the 1998 Gothenburg discotheque fire, the 2017 Stockholm terrorist attack and the mass shooting at a school in Örebro 2025 [4]. More recently, extreme weather events, such as heavy rains and floods, caused significant damage, including the derailment of a passenger train [5]. Additionally, Swedish authorities have issued warnings about an elevated risk of terrorist attacks [6]. Beyond these events, incidents like major road accidents, chemical exposures in industrial environments, and other mass casualty situations may require healthcare professionals to adjust their training accordingly. Therefore, basic competence in disaster medicine is crucial for all medical doctors in Sweden and globally.

International frameworks such as the Sendai Framework for Disaster Risk Reduction and WHO’s Emergency Medical Teams (EMT) initiative have further emphasized the need for structured training and preparedness among healthcare professionals [7]. Globally, several institutions have developed structured training programs in disaster medicine, highlighting the growing recognition of the field’s importance in medical education. One notable example is CRIMEDIM (Research Center in Emergency and Disaster Medicine) in Italy, which has provided postgraduate training through the European Master in Disaster Medicine (EMDM) program [8]. The CRIMEDIM model integrates online learning, residential training, and simulation-based education and has influenced curriculum development in multiple countries. Similar initiatives can be found in Germany [9], and the United States [10], each adapting disaster medicine teaching to their local contexts. These programs demonstrate the feasibility of both standalone and integrated approaches and offer valuable benchmarks for evaluating undergraduate integration in countries like Sweden.

Sweden was among the first countries to introduce disaster medicine education for undergraduates, introducing a one-week course for medical and nursing students in the 1970 s [11]. However, this course was later discontinued to accommodate other subjects, and disaster medicine is no longer a required component in Swedish medical programs, as determined by the Swedish Higher Education Authority. A recent study found that most Swedish medical students rate their knowledge of disaster medical topics as inadequate [12]. Similar findings have been reported in the Netherlands [13], the United States [14], and Germany [15]. The Swedish Ministry of Health and Social Affairs has recommended that disaster medicine to become a mandatory part of the medical program curriculum [11], and the Swedish National Board of Health and Welfare has recently published guidelines for both basic and in-service disaster medicine training for healthcare professionals [16]. However, disaster medical content in Swedish medical programs has not been described in the scientific literature in recent years.

This study aims at providing an overview of the type and extent of disaster medical content currently offered in Swedish medical programs, as well as plans for its inclusion in the revised medical curriculum.

## Method

### Study Design

This study was designed as a sequential qualitative study comprising two phases. In the first phase, a qualitative curriculum mapping of disaster medicine content was conducted through a manual review of course syllabi from all seven Swedish medical programs. This process involved interpretive analysis to identify whether content aligned with national learning objectives for disaster medicine. Although a structured matrix was used to support comparison, the analysis did not involve frequency counts, statistical summaries, or objective coding procedures, and is therefore best described as qualitative.

In the second phase, semi-structured interviews were conducted with program directors and educators to explore how disaster medicine is taught across institutions. Findings from the syllabus mapping informed the interview guide development, allowing for a structured exploration of themes emerging from the curriculum review.

The aim was to explore disaster medical content in undergraduate medical programs at Swedish universities. The Swedish Ethical Review Authority approved the study (approval no. 2023-00774-01). Clinical trial number: not applicable.

### Study setting

Currently, seven universities in Sweden offer medical programs: the University of Gothenburg (GU), Karolinska Institutet (KI), Linköping University (LiU), Lund University (LU), Umeå University (UmU), Uppsala University (UU), and Örebro University (ORU). As of 2021, Sweden is transitioning to a revised six-year medical program. Under the revised program, students will receive their medical license upon graduation, as opposed to after completing an internship, which is the current procedure. The restructuring of medical education in Sweden is influenced by the Bologna Process and its associated reforms across the European Higher Education Area (EHEA), which aim to harmonize academic degrees, promote mobility, and ensure quality assurance in higher education [17].The reorganization of the Swedish medical program into a six-year, cohesive degree aligns with these principles and facilitates integration of transversal competencies such as disaster medicine, which span clinical, organizational, and societal dimensions.

The 5.5-year medical program, which is being phased out, has been described in detail by Lindgren et al. [18]. Currently, each university designs its own curriculum under the supervision of the Swedish Higher Education Authority. In 2018, the Ministry of Education and Research proposed suggestions for the revised medical program, emphasizing scientific and professional competence, as well as medical decision-making, more strongly than in the past [19].

Both phases of this study were conducted in the spring of 2023.

### Review of syllabi

In the first part of this study, syllabi from all Swedish medical programs were compared against a list of suggested disaster medical learning objectives for doctors and nurses, provided by the Swedish National Board of Health and Welfare [16]. This list outlines essential skills and knowledge, though it leaves the decision of whether they should be part of basic education to other departments. Given the ongoing transition from the 5.5-year to the revised 6-year medical program, the curricula for the revised programs are not yet finalized. Therefore, this study only examined syllabi from the 5.5-year program, offering a snapshot of the current state of medical education.

The latest versions of 221 syllabi from the seven Swedish medical programs were downloaded from the universities’ websites. A table was constructed with columns for each university and rows for each learning objective recommended by the National Board of Health and Welfare [16]. Learning objectives and course content descriptions were manually reviewed, and any objective or description that closely matched the suggested learning objectives was included in the corresponding row and column. To be included, they had to be similarly worded to those listed by the National Board of Health and Welfare or cover the same subject matter. The table was then revised to ensure only relevant objectives and descriptions were included. For instance, basic cardiopulmonary resuscitation (CPR) was initially included, but later excluded for not being specific enough to correspond to the recommended disaster medical objectives.

In addition to reviewing the syllabi manually, a keyword search was conducted using terms related to disaster medicine, that facilitated the identification of educators responsible for courses and semesters potentially covering disaster medicine, and the selection of interviewees and informing the content of interview questions. The sequential structure of this study was designed to ensure that the syllabi analysis provided an overview, which subsequently inspired interview questions for the second part of the study. The results are presented in Table 1.

**Table 1:**
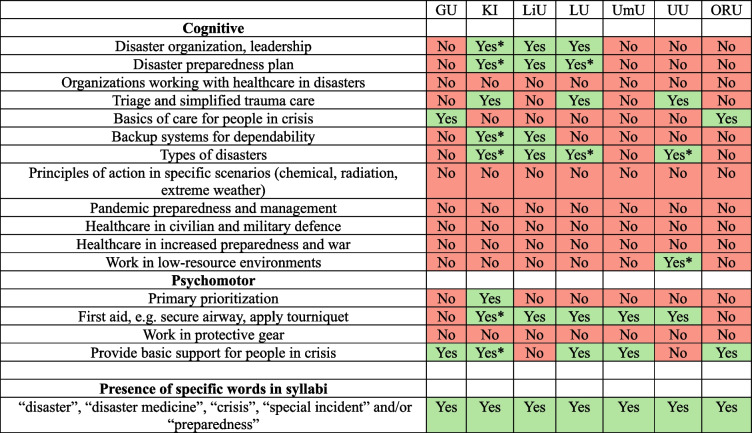
Summary of syllabi

*Relevant learning objective is only included in elective courses

### Participants and recruitment

The second part of this study involved semi-structured interviews to gain deeper insights into disaster medical education in Swedish medical programs. While the syllabi provided an overview, the interviews sought detailed descriptions of course content, teaching methods, and perspectives on disaster medical education. Interviewees included educators and/or members of the medical programs’ boards of education. Educators were recommended by the program directors or identified through professional contacts in disaster medicine. The syllabi analysis also guided the identification of relevant courses, semesters, and potential interviewees.

Potential interviewees were contacted using contact information found on university websites. The interviews were made by means of video calls, by the same researcher who analyzed the syllabi, and were then manually transcribed.

### Interviews

The second part of this study consisted of a qualitative interview study involving key informants from all seven Swedish medical programs. Participants were selected through purposive sampling, targeting individuals involved in curriculum development or responsible for disaster medicine content at each of the seven Swedish medical programs. Invitations were sent by email, with information on the study’s purpose and voluntary participation. In total, 23 individuals were invited, of which 13 participated in interviews.

The interview guides were developed by the first and last authors (SO, YR) and are included as Appendix I and II. They were informed by themes emerging from the syllabus review as well as national disaster medicine education guidelines. The interviews were semi-structured, allowing for both consistent coverage and in-depth exploration of specific topics. Educators received more detailed questions about educational format than program directors.

All interviews were conducted during March–April 2023, by the first author (SO) via secure video conference,. Interviews were audio-recorded and transcribed verbatim. The duration ranged from 17 to 39 minutes (mean: 25 minutes), yielding approximately 100 pages of transcribed material. No translation was required at the data collection stage as all interviews were conducted in Swedish. English quotations presented in this manuscript were translated by the first author (SO), a Swedish-speaking medical student. Care was taken to preserve the tone, intent, and contextual meaning of each quotation.

Data were coded manually and managed using Excel (Microsoft). Initial codes were developed based on the interview guide and revised iteratively. An example of the coding framework is available in Supplementary Appendix III.

To enhance credibility, all interview participants were invited to review the preliminary results (member checking). Eight individuals responded with feedback, leading to minor clarifications and improved phrasing in the category descriptions. This process is described further in the Limitations section.

Reflexivity was addressed throughout the study process. The first author (SO), while being a medical student, did not have prior personal or professional relationships with the interviewees, which contributed to an open and neutral interview climate. To reduce interpretive bias, the research team (comprising researchers with disaster medicine, pedagogy, and qualitative methods expertise) engaged in regular discussions during the coding and analysis stages. Discrepancies in interpretation were resolved collaboratively to enhance trustworthiness.

### Analysis of interviews

After transcription, the interview texts were analysed using qualitative content analysis, as outlined by Graneheim and Lundman [20]. A deductive approach was employed to analyse the manifest content. Two main categories were established: “old/current medical program” and “new medical program.” Additionally, subcategories were created, corresponding roughly to the interview questions, such as “educational methods.” Each interview was read multiple times, and its content was condensed into codes. These codes were then sorted into the appropriate subcategories and categories. If any codes did not fit into the predetermined subcategories, additional subcategories were created to ensure that every code was placed into an exclusive subcategory.

The insights gained from the analysis were summarized in text and diagrams. To ensure the reliability of the analysis, these insights were continuously compared to the original research aim. Furthermore, as described above, all interviewees were invited to review the results and provide feedback on the analysis and findings.

## Results

### Syllabi

The total of 221 syllabi refers to all publicly available course syllabi in the 5.5-year medical programs at the seven Swedish medical faculties at the time of data collection. These syllabi represent official course-level documents that outline intended learning outcomes, teaching methods, and assessment forms. They do not include individual lecture outlines or informal instructional materials. Syllabi were collected from university websites between February and March 2023.

The results from the syllabus review are presented in Table 1. The term “disaster” appears in at least one course syllabus at three out of the seven medical programs: Karolinska Institute (KI), Linköping University (LiU), and Uppsala University (UU). Additionally, Lund University (LU) includes learning objectives related to disaster medicine. For example, in the course *Clinical Medicine 5*, taught during the eleventh semester, one objective is to “identify situations in emergency care where the available resources are insufficient and request appropriate quantitative and/or qualitative reinforcement” [21].

### Interviews

A total of 23 persons were invited to participate in the interviews. Two of them no longer held the position in question, seven declined or were unable to participate, and one did not reply. Of the thirteen who were interviewed, four were representatives of their medical programs’ boards of education and nine were educators with responsibility for the subject of disaster medicine. All of the Swedish medical programs were represented.

**Fig. 1:**
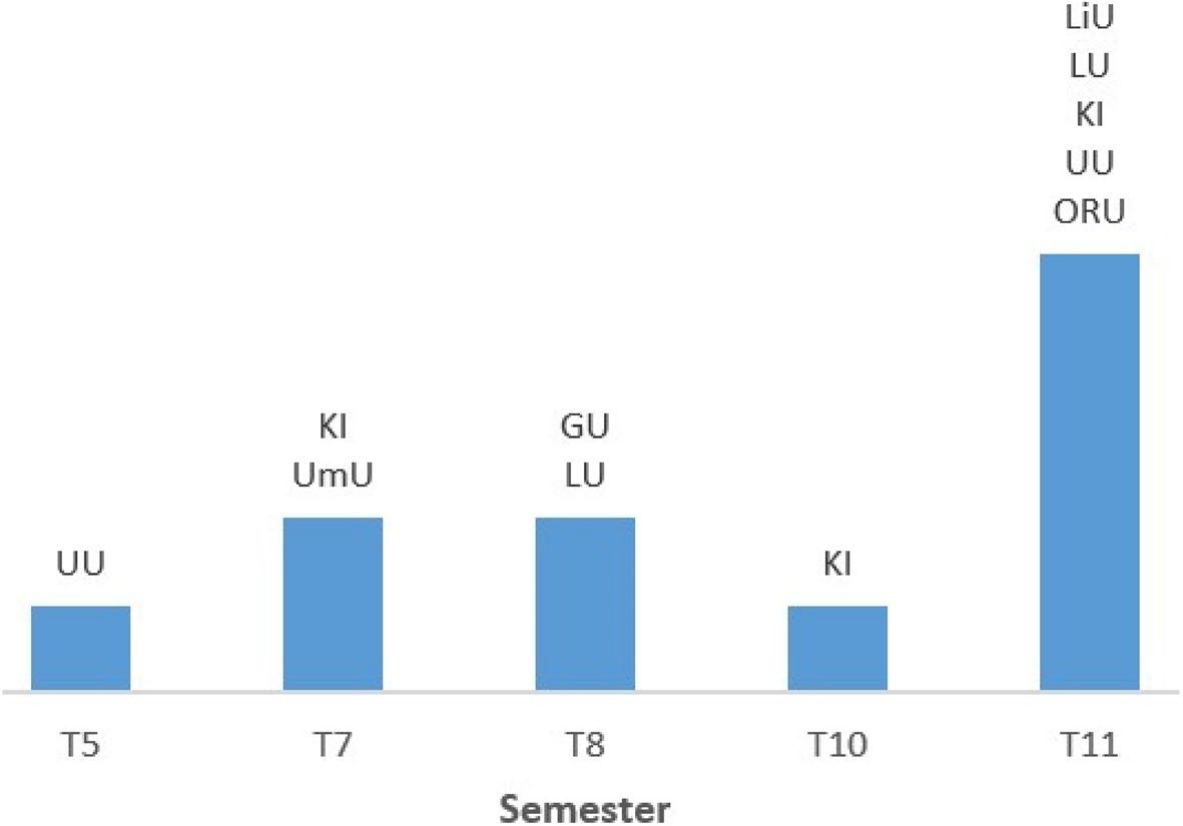
Semesters during which disaster medicine is taught at Swedish medical programs

All seven universities incorporate disaster medical education into their 5.5-year medical programs. At Karolinska Institute (KI), Lund University (LU), and Uppsala University (UU), disaster medicine is included in more than one semester, and most universities teach the subject primarily during the final semester, as illustrated in Fig. 1. KI and LU also offer elective courses in disaster medicine, lasting two and five weeks, respectively [22, 23].

The total number of hours dedicated to disaster medicine varies significantly across universities, making comparisons difficult. This variation is partly due to differences in educational methods and possibly in terminology. Skills relevant to disaster medicine may be taught as part of other subjects, making it harder to recognize them as part of disaster medical education. At Umeå University (UmU), disaster medicine is taught through online self-study, complicating efforts to estimate how much time students spend engaging with the material.

Table 2 summarizes the educational methods used across different universities. At both Linköping University (LiU) and Umeå University (UmU), students are divided into four cohorts during the clinical phase of their medical program. These cohorts are assigned to hospitals in various cities, meaning that most lectures and training sessions are conducted by local educators. UmU has opted for a fully digital approach to disaster medicine education, offering self-study materials, including videos and reading resources, available online. Students are required to complete a mandatory online test as part of this course. LiU takes a slightly different approach by providing online lectures for half a day, followed by in-person training with local supervisors in the afternoon. As part of this, students use the Emergo Train System (ETS), a simulation tool designed to practice the management of emergencies and disasters.

**Table 2:** Summary of educational methods used to reach disaster medical teaching goals in the 5.5-year program in medicine

**University**	**Educational method(s)**
Gothenburg	Lecture.
Karolinska	Lectures. Online self-study and assignments. In-person and digital exercises, cases, and group discussion.
Linköping	Online lecture (live). ETS training in groups, at four different locations.
Lund	8^th^ semester: online lectures (recorded) and self-study, digital test. 11^th^ semester: interactive lecture.
Umeå	Online lectures (recorded) and self-study. Digital test.
Uppsala	Theoretical education followed by: Case discussions to practice triage, prioritization. Board game focused on prioritizing resources in the emergency room. Case discussion about an elderly patient in an overwhelmed emergency room. Clinical skills practice at a clinical training center, immobilization practice. On-site disaster drill (mass casualty incident).
Örebro	Introductory lecture. Roleplay with a simulated bus accident (table-top/simulation hybrid). Self-study assignment: study the disaster preparedness plan of clinical placement hospital, discuss at in-person seminar.

Uppsala University (UU) offers a hands-on disaster exercise at a training site that includes a bus, a train car, and two wrecked vehicles. During this exercise, students take turns acting as both victims and healthcare providers, managing the situation alongside a local ambulance team. UU also integrates a board game, case discussions, and clinical skills practice into their disaster medical education, which is closely linked with their emergency medicine curriculum.

The most frequently mentioned disaster medical topics covered in medical programs include disaster principles, organizational structure, triage, preparedness, global health, mass casualty events, initial assessment and treatment, and real-life events from recent Swedish history. The connection between emergency medicine and disaster medicine is particularly emphasized at UU and Örebro University (ORU).

At UmU, the digital curriculum is divided into five sections: organization and leadership at a disaster site; the role of healthcare during disasters; triage; case studies of past disasters (e.g., fires and traffic incidents); and guidelines for contributing effectively during a disaster.

Although plans for the revised medical programs are still being finalized at all universities, all seven institutions plan to include disaster medicine in their new curricula. Preliminary outlines for these plans are summarized in Table 3. As of the time of the interviews, there is no formal collaboration between universities for the development of new syllabi or teaching materials in disaster medicine, though some educators maintain informal contact with each other.

**Table 3:** Preliminary plans for disaster medicine in the revised 6-year medical program

**University**	**Preliminary plans for the revised medical program**
Gothenburg	Increase, possibly to 1 whole day in 12^th^ semester.
Karolinska	Likely similar to current concept time- and content-wise.
Linköping	Not specified.
Lund	Disaster medicine in 11^th^ or 12^th^ semester, possibly increase time-wise.
Umeå	Likely similar to current concept, in 7^th^ semester.
Uppsala	Likely similar to current concept, with short introduction in 5^th^ semester and main segment in 11^th^ semester.
Örebro	Likely to keep a section in 11^th^ semester but possible increase and incorporation of disaster medicine in earlier semesters to enable progression.

All interviewees agree that competence in disaster medicine is essential for medical students. However, many acknowledge that achieving advanced knowledge in the subject during medical school is challenging. This is due in part to the limited space in the curriculum, but also because teaching the practical application of disaster medicine to students who lack experience in routine medical practice is difficult. Both educators and medical program directors suggest that while medical programs should introduce disaster medicine, most in-depth education and training in the field should occur at the postgraduate level.

A recurring theme in the interviews was the lack of time and resources, with practical disaster drills cited as the most difficult aspect to organize. One educator also highlighted that disaster medicine is often less visible than other subjects, as there are few representatives of the field within clinical settings.

## Discussion

The study revealed a general recognition among university teachers and program directors of the importance of disaster medical content in undergraduate programs. However, the extent and methods of disaster medical education differ significantly across Swedish medical programs. While disaster medicine is part of the curriculum in all seven programs, it is not explicitly mentioned in all syllabi. The time allocated for disaster medicine varies, ranging from a few hours to several days, but comparisons are complicated by differences in terminology and educational methods. Similar variations have been observed in the U.S., where disparities in disaster medical education are also noted [24].

Swedish medical students have reported a median of 2 hours of disaster medical education [12]. A correlation was observed between more self-reported hours and higher self-assessed knowledge, suggesting that around 40 hours would be sufficient to achieve the desired competency level. This mirrors the one-week course previously offered in Swedish medical programs [11], but this study’s interviews suggest that such an increase may be difficult to implement across all universities.

Internationally, incorporating disaster medicine into curricula has been challenging. For example, a German curriculum introduced in 2010 with 28 hours of disaster medicine was poorly implemented [25], and similar difficulties were noted in U.S. programs [15, 26]. Extracurricular courses could serve as alternatives or supplements to university-based education, but no large-scale initiatives exist in Sweden [12]. Medical students in Sweden, the Netherlands, and the U.S. have reported insufficient self-rated knowledge of disaster medicine, particularly in areas such as chemical, biological, radiological, and nuclear (CBRN) events [13–15]. However, the extent of disaster medical training often depends on current events and national priorities, as demonstrated by the increase in disaster medicine education in Fukushima, Japan, following the 2011 nuclear disaster [27].

There is broad consensus in the literature that medical students receive insufficient education in disaster medicine. Interviewees highlighted the difficulty of providing practical training, but Uppsala University (UU) managed to organize a disaster drill, while other universities employed alternative strategies for hands-on learning. Educators’ professional backgrounds also influence the type of education offered. For instance, UU and Örebro University (ORU) align disaster medicine closely with emergency medicine, while Lund University (LU) focuses more on general preparedness and disaster management. These different approaches reflect the absence of national guidelines for disaster medicine education in Swedish medical programs. The Swedish National Board of Health and Welfare has emphasized that a standardized curriculum could be beneficial, particularly as disaster management may require coordination across regional borders [16]. However, there is no international consensus on what constitutes essential content in disaster medical education [28].

The variation in disaster medicine content and teaching approaches observed in Swedish medical programs mirrors findings from studies in other countries. International literature highlights a global inconsistency in how, when, and to what extent disaster medicine is taught in undergraduate education [25, 29]. For example, while some countries integrates disaster preparedness in both public health and clinical courses due to frequent natural hazards [30], other countries such as Italy rely more on postgraduate or elective training models [8]. Our findings suggest that Swedish educators, like their counterparts elsewhere, recognize the importance of disaster medicine but face structural and pedagogical barriers to implementation. These include limited curricular space, lack of national standards, and insufficient faculty training.

The interviews revealed that some Swedish programs are attempting to embed disaster-related competencies through interdisciplinary simulations or thematic modules. Such efforts resonate with best practices described in other contexts, where combining cognitive knowledge with experiential learning has shown positive outcomes. However, the absence of shared national learning objectives or evaluation tools in Sweden risks perpetuating inequities in student preparedness. A future opportunity lies in aligning Swedish efforts with international competency frameworks such as the WHO EMT standards or the EMDM core curriculum, to facilitate benchmarking, exchange, and standardization.

Another area for discussion is whether disaster medicine should be integrated with other subjects, such as emergency medicine, or recognized as a distinct subject with designated representatives at each university. Although disaster medicine is not mentioned in all syllabi, this does not necessarily mean it is not taught. However, excluding it from official documents creates ambiguity about the specific learning objectives. Clearly defined learning objectives in the syllabi would help ensure that disaster medicine education is both developed and protected within the broader medical curriculum.

Determining what disaster medicine competencies are realistic and appropriate for undergraduate medical education requires careful consideration of students’ clinical experience, available teaching time, and the complexity of required skills. Based on the analysis of course content, interviews with educators, and national recommendations from the Swedish National Board of Health and Welfare [31], we suggest that the undergraduate level should focus on foundational cognitive and procedural competencies. These include understanding the principles of healthcare organization and leadership during major incidents, knowledge of triage and simplified treatment strategies in mass casualty situations, familiarity with national preparedness plans, and the ability to perform basic life-saving interventions such as haemorrhage control and airway management. Additionally, students should be introduced to psychological first aid and ethical aspects of care under resource constraints. These outcomes align with both the national educational recommendations and Bloom’s taxonomy at the lower-to-intermediate cognitive levels (knowledge, comprehension, application). More advanced skills—such as managing CBRN scenarios or inter-agency command roles—are better suited for postgraduate or continuing education settings. Clear documentation of these undergraduate outcomes can be achieved through integrated simulations, scenario-based evaluations, and structured reflections, ensuring that disaster medicine education remains pedagogically sound and contextually realistic.

To effectively achieve these learning objectives, medical programs can employ a variety of educational methods that support both knowledge acquisition and experiential learning. Didactic approaches such as interactive lectures and flipped classroom models can introduce theoretical concepts, while case-based learning can contextualize disaster scenarios and decision-making. Active methods—including tabletop exercises, role-playing, and low-fidelity simulations—offer students opportunities to apply principles of triage, ethical reasoning, and teamwork in a controlled environment. Where resources allow, higher fidelity simulation or interdisciplinary exercises may further enhance preparedness.

A recurring theme in the interviews was the challenge of organizing large-scale practical disaster drills, primarily due to time, staffing, and logistical constraints. While high-fidelity simulations can offer valuable experiential learning, they are not strictly necessary to achieve educational objectives in disaster medicine. Tabletop exercises, virtual simulations, and case-based discussions have been shown to effectively support the development of situational awareness, decision-making, and ethical reasoning under pressure [28]. These low-resource methods are particularly well suited to undergraduate education, where time and infrastructure may be limited. Several interviewees reported using scenario-based discussions and interprofessional tabletop formats to introduce core principles of disaster response.

Assessment of student achievement should be aligned with these methods. Written examinations and structured oral assessments can test factual knowledge and reasoning. Reflective essays and group reports allow for deeper analysis of ethical and psychological dimensions, while Objective Structured Clinical Examinations (OSCE) or scenario-based practical assessments can evaluate hands-on skills and communication under pressure. A combination of these strategies ensures comprehensive evaluation of both cognitive and procedural competencies within the scope of undergraduate medical education.

A coordinated national effort is essential to ensure that disaster medicine education in Sweden aligns with evolving healthcare needs and societal risks. In this context, several key stakeholders could assume leadership roles in standardizing disaster medicine content and learning outcomes. The Swedish National Board of Health and Welfare, which has already developed recommended disaster medicine competencies [31], is well-positioned to provide national guidance and benchmarking. In parallel, the Swedish Society of Medicine, the Swedish Medical Association and the Association of Swedish Higher Education Institutions could facilitate collaboration between universities, ensuring integration with existing curricular frameworks and professional expectations. To operationalize this effort, we propose the establishment of a national task force for disaster medicine education, composed of representatives from universities, clinical experts, educational researchers, and national authorities. Such a platform could develop consensus-based core competencies, suggest scalable didactic models, and promote shared educational resources—thereby supporting both equity and quality in undergraduate disaster medicine education across Sweden.

### Limitations

This study relies on qualitative data, which involves interpretation. The table of learning objectives was created by one researcher, and although revised carefully, another researcher may have produced slightly different results. Additionally, syllabi do not detail every lecture and exercise, making it difficult to assess the full scope of disaster medicine education. The fact that disaster medicine is not explicitly mentioned in some syllabi does not mean it is absent from the curriculum. Thus, the syllabus review should be seen as part of a larger context.

An additional limitation is that the newly implemented six-year medical curricula were still under development at the time of data collection. Although all programs had published core structural information, detailed course-level syllabi and finalized disaster medicine content were in many cases not yet available. This may have led to an underestimation of planned or upcoming content, particularly for cohorts that had not yet reached clinical semesters.

Interviews are another method requiring interpretation, both in terms of how questions are posed and how answers are analysed. Although the interview guide helped standardize questions, the interviewer may have influenced responses. Furthermore, while interviewing educators and education board representatives provided valuable insights, only four board members were interviewed, limiting the breadth of this perspective. To mitigate misunderstandings, all interviewees were invited to review the interview results and provide feedback.

This paper references several studies in which students have assessed their own knowledge and abilities. While self-assessed knowledge levels among medical students provide valuable insights into perceived preparedness, such measures have inherent limitations. Self-assessment may not accurately reflect actual competency, particularly in complex domains like disaster medicine that require both theoretical understanding and practical decision-making skills. Studies have shown that students can overestimate or underestimate their abilities depending on prior exposure, confidence, or the specificity of questions asked [32]. To complement subjective assessments, future evaluations of disaster medicine education should include objective tools, such as written examinations, structured clinical scenarios, or Objective Structured Clinical Examinations (OSCE) tailored to mass casualty or low-resource settings. Incorporating standardized assessments would enable more precise monitoring of educational outcomes and support evidence-based curriculum development.

In line with qualitative research standards, we considered the concepts of credibility, transferability, and dependability to assess the trustworthiness of this study [20]. To ensure credibility, we employed a structured interview guide, provided clear descriptions of the analytical steps, and used member checking to validate the interpretation of results. Five participants provided minor corrections on the preliminary findings, and their feedback was incorporated into the final analysis. Regarding transferability, we provided a detailed description of the study context, including university-specific information, curricular structure, and participant backgrounds. This allows readers to assess whether findings are applicable to other settings.

To support dependability, all interviews were conducted and transcribed by the same researcher using a consistent methodology, and data were analysed using established qualitative content analysis techniques. While researcher interpretation can never be fully neutral, transparency in methods and reflexivity in analysis were maintained throughout the study.

## Conclusions

This study demonstrates that while disaster medicine is taught at all Swedish medical schools, there are significant variations in its content, scope, and delivery. The findings highlight that disaster medicine is often inconsistently integrated, sometimes absent from syllabi, and generally lacks national coordination. Despite strong support among educators for including disaster medicine in undergraduate training, challenges such as limited curricular time, lack of standardized learning objectives, and scarce practical training opportunities persist.

Educators across all institutions emphasized the need for disaster medicine to remain in the revised six-year curriculum, with several planning to expand its scope. However, without a coordinated national approach, disparities between programs are likely to continue. Establishing shared core competencies, improving educational methods, and fostering collaboration between universities are essential steps to ensure that future physicians are adequately prepared to respond to mass casualty incidents and other crises.

## Supplementary Information

Supplementary Material 1.

## Data Availability

The datasets generated and/or analyzed during the current study are not publicly available due to ethical restrictions but are available from the corresponding author (Yohan Robinson, yohan.robinson@gu.se) on reasonable request.

## Abbreviations

CBRN: Chemical, Biological, Radiological, and Nuclear
EMDM: European Master in Disaster Medicine
EMT: Emergency Medical Teams
ETS: Emergo Train System
GU: University of Gothenburg
KI: Karolinska Institutet
LiU: Linköping University
LU: Lund University
ORU: Örebro University
UmU: Umeå University
UU: Uppsala University
WHO: World Health Organization
