# Supplementary material for: Disaster medicine in Swedish undergraduate medical education: analysing current programs and future integration in the six-year curriculum
Source: BMC Med Educ. 2025 May 20;25:731. doi: 10.1186/s12909-025-07324-2 (PMC12093758; doi:10.1186/s12909-025-07324-2)
Supplement: Supplementary file 1 — Supplementary Material 1. [file 12909_2025_7324_MOESM1_ESM.docx]

**Appendix I.** Interview guide: program directors.

The interview relates to the disaster medical education at Swedish medical programs. The interviewer is neutral. Begin the interview by introducing yourself and giving a short introduction to the project:

The aim of the study is to investigate the disaster medical education that Swedish medical students have received in recent years, and whether there are plans to change the education as the new six-year medical program is introduced. The investigation consists of interviews with medical program directors as well as educators in disaster medicine.

Confirm that the interviewee has consented to participating and to recording of the interview. Announce when you start recording. Confirm after starting the recording that the interviewee has received written and oral information about the study and consented to participation. Remind the interviewee of their right to abstain from answering questions and to withdraw from the study at any time without specifying a reason.

**Interview questions**

1. Is disaster medicine included in the mandatory education of the 5,5-year medical program at your university?
   1. If YES: in which semester/s is it taught?
   2. If YES: how many hours are assigned to disaster medicine?
2. Do you know if there are plans to include disaster medicine in the mandatory education of the new, 6-year medical program?
   1. If YES: in which semester/s?
   2. If YES: how many hours are assigned to disaster medicine?
3. Have learning objectives and/or syllabi related to disaster medicine been developed for the new, 6-year program?
   1. If YES: how have they been developed?
   2. If NO: is there a plan for how they are to be developed?
4. Do you know whether there are plans to offer a disaster medical elective course/element in the new, 6-year medical program?
   1. If YES: in which semester/s will it be taught?
   2. If YES: how many credits will the course be allocated?
5. Have learning objectives and/or syllabi been developed for a possible elective course in disaster medicine in the new, 6-year medical program?
   1. If YES: how have they been developed?
   2. If NO: is there a plan for how they are to be developed?
6. Does your university collaborate in any way with other Swedish universities in the development of learning objectives and teaching material related to disaster medicine for the new, 6-year medical program?
   1. If YES: what does that collaboration look like?
7. What is your perception of the need for disaster medical education in Sweden?
8. Can you give one or more examples of situations which a newly graduated doctor might have to handle in which disaster medical knowledge could be of use?
9. Is there anything you would like to add as regards the disaster medical education in the medical program?

The above questions are to be included in every interview. Depending on the answers given by each interviewee, and on university-specific results from the reading of syllabi, other questions may be added.

**Finish the interview**

Announce that the interview is over and stop the recording. Thank the interviewee for their participation. Explain how they can reach you in case of any questions.

**Appendix II.** Interview guide: educators in disaster medicine.

The interview relates to the disaster medical education at Swedish medical programs. The interviewer is neutral. Begin the interview by introducing yourself and giving a short introduction to the project:

The aim of the study is to investigate the disaster medical education that Swedish medical students have received in recent years, and whether there are plans to change the education as the new six-year medical program is introduced. The investigation consists of interviews with medical program directors as well as educators in disaster medicine.

Confirm that the interviewee has consented to participating and to recording of the interview. Announce when you start recording. Confirm after starting the recording that the interviewee has received written and oral information about the study and consented to participation. Remind the interviewee of their right to abstain from answering questions and to withdraw from the study at any time without specifying a reason.

**Interview questions**

1. How are you involved in the disaster medical education at your university?
2. Is disaster medicine included in the mandatory education of the 5,5-year medical program at your university?
   1. If YES: in which semester/s is it taught?
   2. If YES: how many hours are assigned to disaster medicine?
3. Have there been major changes to the syllabus concerning disaster medicine in the past five years?
   1. If YES: what type of changes have been made?
4. Is disaster medicine allocated its own teaching hours at your medical program (e.g. in the form of a lecture solely dedicated to disaster medicine, rather than being part of a lecture on emergency medicine)?
   1. If NO: within which other subject is disaster medicine taught?
5. Which aspects of disaster medicine are included in the disaster medical education? (The answer may contain more or less detail).
6. Which educational methods are used for the subject of disaster medicine? E.g. traditional lectures, skills practices, or online teaching?
7. Is there an elective disaster medical course at your medical program?
   1. If YES: in which semester/s is it taught? How many credits does it comprise?
   2. If YES: how many students can take the elective/how many students took the elective this past semester?
8. Do you know if there are plans to include disaster medicine in the mandatory education of the new, 6-year medical program?
   1. If YES: in which semester/s?
   2. If YES: how many hours are assigned to disaster medicine in total?
   3. If YES: do you know how syllabi and learning objectives for disaster medicine will be developed, if they have not been developed already?
   4. If YES: are you aware that the Swedish Board of Health and Welfare recently published suggested syllabi for disaster medical education and training? Are these used at your medical program, e.g. as inspiration for new learning objectives?
9. Are there plans to offer a disaster medical elective course/element in the new, 6-year medical program?
   1. If YES: in which semester/s will it be taught?
   2. If YES: how many credits will the course be allocated?
10. Is there anything you think should be included in the disaster medical education in the new, 6-year medical program, which is not included in the current program?
11. What do you think is the best method for teaching disaster medicine?
12. What is your perception of the need for disaster medical education in Sweden?
13. Can you give one or more examples of situations which a newly graduated doctor might have to handle in which disaster medical knowledge could be of use?
14. Should disaster medicine be included in the basic training of medical doctors?
15. Is there anything you would like to add as regards the disaster medical education in the medical program?

The above questions are to be included in every interview. Depending on the answers given by each interviewee, and on university-specific results from the reading of syllabi, other questions may be added.

**Finish the interview**

Announce that the interview is over and stop the recording. Thank the interviewee for their participation. Explain how they can reach you in case of any questions.

Appendix III – Interview Analysis: Excerpt from Coding Framework and Illustrative Quotes

**Overview of Coding Framework**

| **Main categories** | **Subcategories** | **Example Quote** |
| --- | --- | --- |
| 1. Old/current  (5,5 year) medical program | Disaster medicine in the current curriculum | “There is a mandatory day [which means] if you are not present you have to do an assignment to make up for it.” |
|  | Educational methods | “We have integrated some practical exercises into our scenarios […], a bit more equipment in the station-based exercises.”  “Purely traditional lectures, with some questions and interaction, [followed by] a practical exercise.” |
|  | Disaster medical content | “Organization and management at the accident site, the role of healthcare, a bit about triage, and then there are examples of disaster medicine [scenarios] and how one can contribute.” |
|  | Difficulties in disaster medical education | “It’s an incredibly large field to try and cover in a single day.”  “You can’t get very far at undergraduate level, [the students have] so little clinical experience at that point.” |
| 2. New (6 year) medical program | Perception of need for disaster medicine at undergraduate level | “I think it’s important to get an introduction to these topics.”  “I think it should be included in the medical program, not least now that doctors receive their license upon graduation from the 6-year program.” |
|  | Suggestions or wishes related to disaster medicine in the new program | “To start earlier, [so that] students have time to progress, to get basic knowledge early on which can be expanded, and then move on to a more advanced level later”  “What we have now is almost nothing. So I think we should definitely have some form of expansion.” |

**Supplementary Table 1**. Overview of Included Swedish Medical Programs

| **University** | **City** | **Annual Student Intake** | **Curriculum Characteristics** | **Disaster Medicine Integration (from interviews)** |
| --- | --- | --- | --- | --- |
| University of Gothenburg (GU) | Gothenburg | ~380 students | Traditional block system, early clinical exposure. | Some content in psychosocial care; no formal disaster module. |
| Karolinska Institutet (KI) | Stockholm | ~431 students | Integrated curriculum, emphasis on public/global health. | Multiple elements in theory and skills; planning expansion. |
| Linköping University (LiU) | Linköping | ~340 students | Problem-based learning (PBL), longitudinal themes. | Thematically integrated; planned expansion under new curriculum. |
| Lund University (LU) | Lund | ~330 students | Modular structure with increasing clinical responsibility. | Disaster medicine included in emergency medicine course. |
| Umeå University (UmU) | Umeå | ~359 students | Regional campus model, strong rural medicine component. | Limited content; some training in remote emergency care. |
| Uppsala University (UU) | Uppsala | ~280 students | Organ system-based curriculum; early exposure to clinical skills. | Disaster medicine included in elective course; one instructor engaged. |
| Örebro University (ORU) | Örebro | ~210 students | Newer program; strong interprofessional and simulation-based learning. | Disaster concepts integrated in simulation training; no specific course. |

Note on annual student intake: Swedish medical programs welcome new students twice a year, with one cohort starting in January and one in August or September. It is common for universities to accept a number of students exceeding the true intake capacity to account for early dropouts; thus, enrolment for the second semester is usually lower than the numbers presented here. Student intake has increased at all Swedish medical programs over time; presented here are the numbers of students accepted into medical school in 2024 according to the Swedish Council for Higher Education (<https://www.uhr.se/studier-och-antagning/antagningsstatistik/>)

**Supplementary Table 3.** Translated Learning Objectives in Disaster Medicine According to Bloom’s Taxonomy

| **Domain** | **Learning Objective (Translated from Socialstyrelsen, 2022)** |
| --- | --- |
| **Cognitive** | Know the principles of healthcare organization and management in disaster areas and healthcare units during special events. |
|  | Understand the structure and application of disaster medical preparedness plans, different levels of preparedness, and use of action cards. |
|  | Know of organizations collaborating with healthcare and their responsibilities during special events. |
|  | Understand principles of prioritization (triage) and simplified treatment methods during events involving injuries caused by physical violence, including high-energy violence such as projectile and blast injuries. |
|  | Know of existing backup systems for healthcare operational reliability (information technology, communication, electricity, and water supply).  Know of different types of events that can affect society and their specific demands on healthcare and personnel.  Know of principles for managing scenarios involving harmful chemicals, ionizing radiation, large fires, and extreme weather conditions.  Understand preparedness for and principles for managing pandemics.  Know of civil and military defense medical organizations and the role of healthcare in total defense.  Know of civil and military defense medical organizations and the role of healthcare in total defense.  Understand principles of working in low-resource areas during natural and famine disasters, and the demands they place on healthcare and personnel. |
| **Psychomotor** | Perform primary triage of victims in disaster areas using prioritization algorithms. |
|  | Provide basic primary treatment measures according to evidence-based "first aid" guidelines, such as manual airway management, recovery position, pressure bandages and tourniquets, and stabilizing of fractures. |
|  | Working in protective equipment when handling infectious and/or contaminated patients |
| **Affective** | Know the basics of crisis support for those affected during special events and support for staff after interventions.  Provide basic crisis support to affected individuals. |
